# Analysis of the Failure Process of Elements Subjected to Monotonic and Cyclic Loading Using the Wierzbicki–Bai Model

**DOI:** 10.3390/ma14216265

**Published:** 2021-10-21

**Authors:** Urszula Janus-Galkiewicz, Jaroslaw Galkiewicz

**Affiliations:** Faculty of Mechatronics and Mechanical Engineering, Kielce University of Technology, 25-314 Kielce, Poland; janus.urszula@gmail.com

**Keywords:** Wierzbicki–Bai model, fatigue, tensile test, stress triaxiality, Lode parameter

## Abstract

This article presents the results of a simulation in which smooth cylindrical and ring-notched samples were subjected to monotonic and fatigue loads in an ultra-short-life range, made of Inconel 718 super alloy. The samples displayed different behaviors as a result of different geometries that introduced varying levels of stress triaxiality and loading methods. The simulations used the Wierzbicki–Bai model, which took into account the influence of stress tensors and stress-deviator invariants on the behavior of the material. The difference in the behaviors of the smoothed and notched specimens subjected to tensile and fatigue loads were identified and described. The numerical results were qualitatively supported by the results of the experiments presented in the literature.

## 1. Introduction

Understanding the processes that occur in structural elements allows one to design more durable parts. The finite element method is currently the basic tool for analyzing the influence of various factors on material behavior. In the case of slowly changing monotonic loads, it seems that we obtain reliable results, but there are still problems with both the calculations and the interpretation of the results for other types of loads. Methods to speed up the calculation time [1] and to take into account various aspects of the loading process, such as dynamic loading [2] and the appearance of cracks [3], which are the final feature of the critical cross-section, are constantly being developed.

A fundamental problem with these methods is the evaluation of the strength of the material. The static tensile test, in many cases, does not give sufficient answers to the question of when the failure will occur. When assessing the strength of elastic–plastic materials, the basic problem is with how to determine the moment of transition into a plastic state. Many models can be used for this; the most popular models are Mises–Huber, Tresca, Drucker–Prager, and Mohr–Coulomb. They can be used with greater or lesser success for various types of materials, although they have their limitations. For example, the Mises–Huber model is not sensitive to stress triaxiality and the Lode parameter, whereas the Drucker–Prager model considers the fact that the material behaves differently under tension and compression, which means that it is sensitive to stress triaxiality. The influence of the Lode parameter can be found in the Tresca and Mohr–Coulomb models. The experiments that were initiated by Wierzbicki and Bai [4], and later conducted by many others, have shown [5,6] that the level of plastic deformation at the moment of failure of the tested element depends on the stress triaxiality and the Lode parameter. On this basis, a new model of plasticity was proposed. Numerous examples of the use of this model have shown very good compliance between the numerical simulations and the experimental results. The model has previously been used for monotonic loads, but recently articles in the field of fatigue (so far, of ultra-low cycles) have started to appear, presenting very reliable results. In [7], the Wierzbicki–Bai model was used to reproduce measurable displacement and force parameters in the range of monotonic tensile or compression tests of samples with different geometries, which can be characterized by the triaxial level and the Lode parameter. The above-mentioned model was used in [8,9] to analyze the fatigue loading process in the range of up to 100 cycles.

The model itself has been gradually developed for several years [10,11,12,13]. The yield criterion was extended to take into account the influence of triaxiality and the Lode parameter, as well as the numerical application and the parameters supporting the calculation process. In the present work, our attention was focused on analyzing the load process of smooth cylindrical specimens and specimens with a ring notch, and tracing the development of the damage in the material, as the accumulated effective plastic strain properly normalized. As a result, the influence of the notch-root radius on the behavior of the specimens that were subjected to a tensile load was identified and described, and the behavior of specimens subjected to a fatigue load was compared with those under a tensile load. All results were verified by experimental data.

The structure of the article is as follows: in the first section, the theoretical background is provided; then, the materials and calculation methods are discussed; in the third section, the achieved results are presented; and in the last section, our results and conclusions are discussed.

## 2. Theoretical Background

Stress triaxiality is understood to mean the ratio of hydrostatic stress to effective stress, that is, the ratio of the first stress tensor invariant by a function that depends only on the second invariant of the deviatoric stress tensor [14].

(1) $$\eta =\frac{\sigma_{m}}{\sigma_{e}}$$

The Lode parameter is slightly more complex to explain [15]. If the greatest tangential stress expressed by principal stresses is $\tau =\left(\sigma_{I}-\sigma_{III}\right)/2$ and the normal stress in the plane of maximum tangential stress is equal to $\sigma_{N}=\left(\sigma_{I}+\sigma_{III}\right)/2$, the Lode parameter can be written by the equation $L=\left(\sigma_{II}-\sigma_{N}\right)/\tau$ [16]. Although it is not visible at first glance, this parameter is related to the third invariant of the deviatoric stress tensor. Let us introduce the parameter denoting the normalized third invariant of the deviatoric stress tensor [17]:

(2) $$\xi =\frac{27}{2}\frac{J_{3}}{\sigma_{e}^{3}}$$

This quantity is related to the Lode angle, determined on the deviatoric plane according to the equation:

(3) ξ=cos(3θ)

and with the Lode parameter *L*:

(4) $$\xi =L\left(9-L^{2}\right)/\sqrt{{\left(L^{2}+3\right)}^{3}}$$

**Figure 1:**
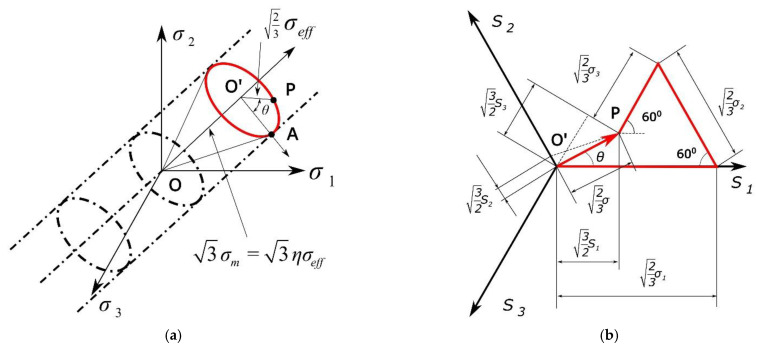
Components of the stress tensor in the principal stress space (**a**). Components of the stress tensor on the deviatoric plane (**b**).

Knowing the value of the effective stress, the Lode angle, and the stress triaxiality, it is possible to uniquely describe the stress state, as shown in Figure 1.

On the deviatoric plane, the yield surface is reduced to a circle (Figure 1a). However, the triaxiality of the stress affects its radius, and the Lode angle affects the position of the point on the circle that defines the load state. Moreover, the Lode angle may influence the shape of the yield surface [12]. It does not necessarily have to be a circle; this shape should be determined and verified experimentally. Wierzbicki et al., therefore, proposed universal functions based on both parameters. This function consists of two terms separated by variables.

The impact of stress triaxiality is expressed by the function:

(5) $$f\left(\eta \right)=1-c_{\eta }\left(\eta -\eta_{0}\right)$$

where *c_η_* is the coefficient of stress triaxiality depending on plasticity, *η*_0_ is the reference value of the stress triaxiality, and *η* is the current value of the stress triaxiality.

The influence of the Lode angle on the shape of the yield surface is more challenging. The function that describes this impact has evolved as new, more complete experimental results have appeared, and finally reached the form:

(6) $$f\left(\theta \right)=c_{\theta }^{s}+\left(c_{\theta }^{ax}-c_{\theta }^{s}\right)\left(\frac{m+1}{m}\right)\left(\gamma -\frac{\gamma^{m+1}}{m+1}\right)$$

where $\gamma =\frac{\cos\left(\pi /6\right)}{1-\cos\left(\pi /6\right)}\left(\frac{1}{\cos\left(\theta -\pi /6\right)}-1\right)$. The values of “*c*” can be treated as material constants, but in the fullest version they are described by functions:

(7) $$c_{\theta }^{ax}=\{\begin{array}{cc} c_{\theta }^{t} & \bar{\theta }\geq 0\\ c_{\theta }^{c} & \bar{\theta }<0 \end{array}$$

(8) $$c_{\theta }^{s}=\sqrt{3}/2+\left(B_{1}e^{-B_{2}\varepsilon_{pl}}\right)f\left(\bar{\theta }\right)$$

(9) $$f\left(\bar{\theta }\right)={\left(1-{\left|\bar{\theta }\right|}^{B_{3}}\right)}^{B_{4}}$$

where *B* parameters are the quantities selected, so that the simulation results are as close as possible to the experimental results. Their task is to consider the influence of large deformations and to correct the influence of the Lode angle. The constants $c_{\theta }^{t}$ and $c_{\theta }^{c}$ allow the distinction between compression and tension.

It should be emphasized that Equation (6), proposed by Wierzbicki, was not the only function used. In the papers inspired by the works of Wierzbicki et al. [16,18], other functions were used based on the parameters ($\sigma_{eff},\;\bar{\theta },\;\eta$), creating the so-called Haigh–Westergaard space.

Taking into account Equations (5) and (6), the plasticity function proposed by Wierzbicki et al. can be written as [7]:

(10) $$\sigma \left(\varepsilon_{pl},\eta ,\theta \right)=\sigma \left({\bar{\varepsilon }}_{p}\right)\left[1-c_{\eta }\left(\eta -\eta_{0}\right)\right]\left[c_{\theta }^{s}+\left(c_{\theta }^{ax}-c_{\theta }^{s}\right)\left(\frac{m+1}{m}\right)\left(\gamma -\frac{\gamma^{m+1}}{m+1}\right)\right]$$

where $\sigma \left({\bar{\varepsilon }}_{p}\right)$ is the effective-stress value read from the tensile diagram presented in the logarithmic strain-true stress system. The terms (m+1)/m and $\gamma^{m+1}/\left(m+1\right)$ have been added to facilitate numerical calculations.

**Figure 2:**
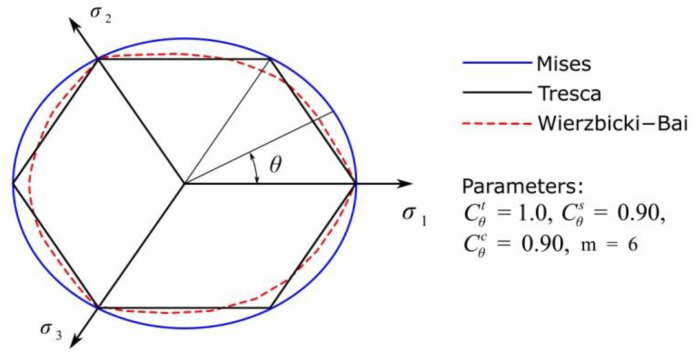
Yield surfaces for various yield criteria.

An example of the results of plasticity surface modification through Equations (5) and (6) is presented in Figure 2.

The fully developed Wierzbicki–Bai model contains many material constants, which can be considered a weakness; however, these constants are easily determinable, and the plasticity function itself can be used in a simplified form.

**Figure 3:**
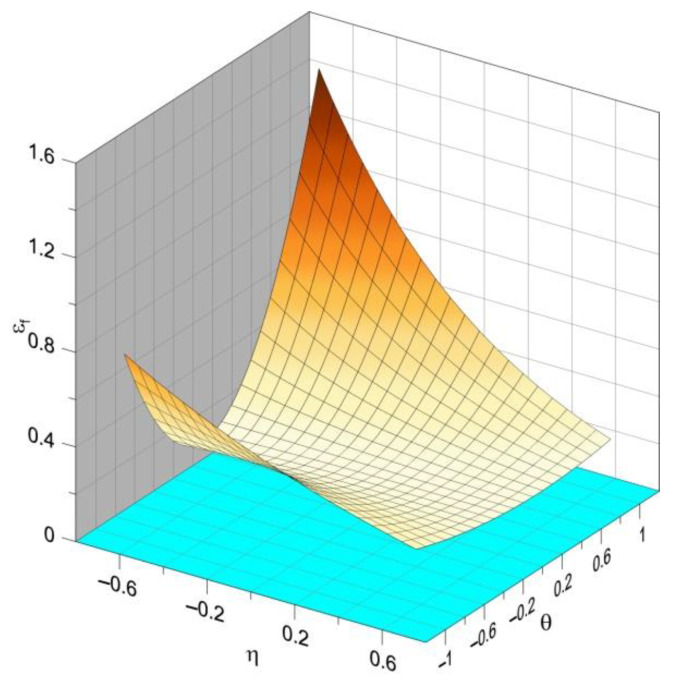
Exemplary fracture locus (source: own simulations).

Research shows that in the case of elastic–plastic metals, both quantities, that is, the Lode parameter and the stress triaxiality, play essential roles when the yield conditions are analyzed. Triaxiality controls the void growth [19,20,21], whereas the Lode parameter is associated with a change in the shape of the growing voids [22,23,24]. As a result, they influence the critical strain, creating a fracture locus. An example diagram showing the dependence of the strain at the critical moment of triaxiality and the Lode angle is shown in Figure 3.

## 3. Material

The material used in the work was Inconel 718 alloy, which is a nickel–chromium alloy characterized by its high resistance to corrosion and creep. The chemical composition of the alloy is presented in are given in Table 1 in [7].

**Figure 4:**
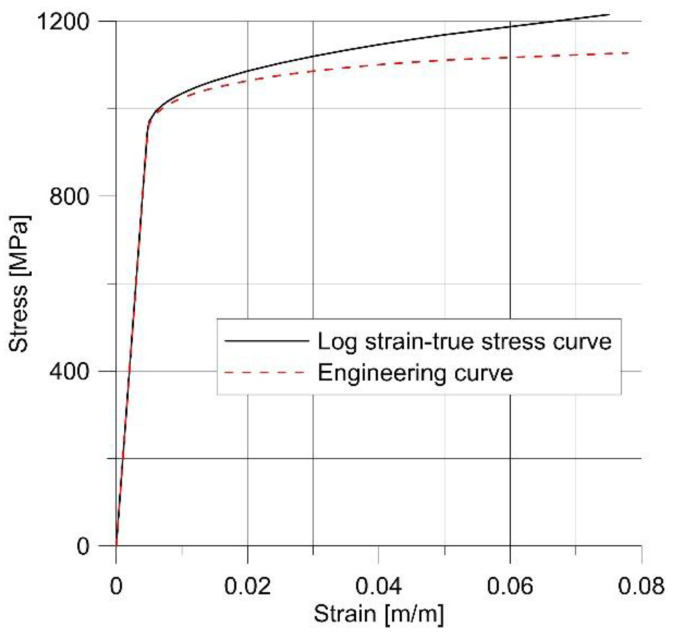
Comparison of engineering and true stress–strain curves (source: own computations).

This material was selected since the parameters required to fully characterize it in the Wierzbicki–Bai model are readily available in the literature, and the process of obtaining them is described in detail in [7]. The tensile curve was reconstructed with the help of Equation (4), and is presented in Figure 4.

After reaching the yield point, the material was described with the Ludwik curve in the form:

(11) $$\sigma \left({\bar{\varepsilon }}_{pl}\right)=\sigma_{0}+K\overset{-n}{\varepsilon_{pl}}$$

The entire true stress–strain curve was represented by a set of material constants: Young’s modulus *E* = 200,000 MPa, Poisson’s ratio ν = 0.284, yield stress *σ*_0_ = 945.1 MPa, and parameters of the Ludwik curve *K* = 835.4 MPa and *n* = 0.425.

However, the numerical calculations required a much wider set of necessary data to determine the yield surface. These constants are given in Table 5 in [7].

It is worth emphasizing at this point that the symmetry of the yield locus concerning tension and compression was adopted.

**Figure 5:**
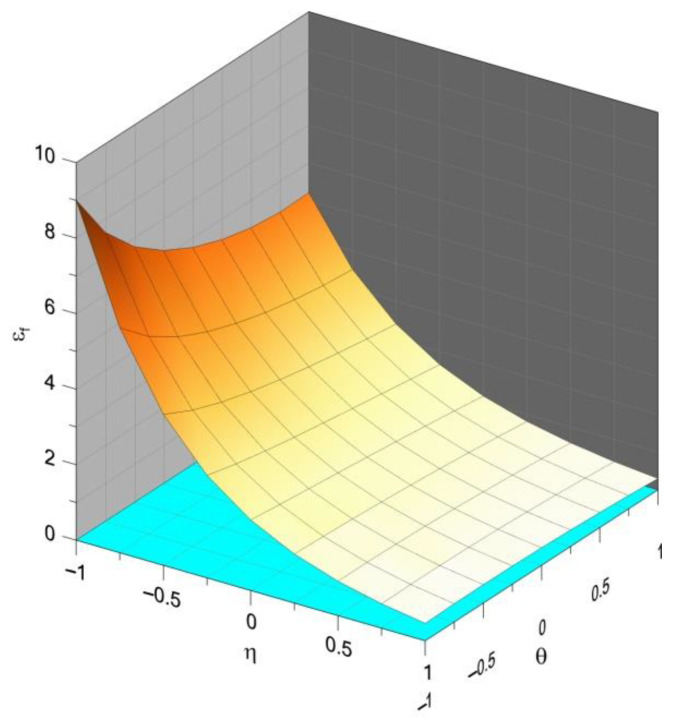
Simplified fracture locus.

Another problem was determining the moment of material failure. The model should follow the experimental data; however, the damage can be modeled in various ways [25]. The parameter D was used for this, and calculated according to the equation:

(12) $$D=\int_{0}^{{\bar{\varepsilon }}_{pl}}\frac{d{\bar{\varepsilon }}_{pl}}{{\bar{\varepsilon }}_{f}\left(\eta ,\bar{\theta }\right)}$$

where ${\bar{\varepsilon }}_{pl}$ is the equivalent plastic strain. The critical strain ${\bar{\varepsilon }}_{f}$ depended on the stress triaxiality and the Lode parameter. Its full analytical form was described in [7]. The easiest way to create it was to fit the experimental data as described in article [26]. The 3D fracture locus in this case was constructed as follows:

(13) $$\begin{array}{cc} \varepsilon_{f}= & \left(N_{1,1}\eta^{2}+N_{1,2}\eta +N_{1,3}\right){\bar{\theta }}^{3}+\\ \left(N_{2,1}\eta^{2}+N_{2,2}\eta +N_{2,3}\right){\bar{\theta }}^{2}+\\ \left(N_{3,1}\eta^{2}+N_{3,2}\eta +N_{3,3}\right)\bar{\theta }+\\ \left(N_{4,1}\eta^{2}+N_{4,2}\eta +N_{4,3}\right) \end{array}$$

where the table of coefficients had the form of Table 1. The fracture locus is shown in Figure 5. To decrease the dynamic effects after crack initiation, an additional “softening” function was introduced:

(14) $$\sigma_{o}=\{\begin{array}{cc} \sigma_{0} & 0\leq D<1\\ \beta \sigma_{0} & 1\leq D\leq D_{c} \end{array}$$

where the softening factor *β* is:

(15) $$\beta ={\left(\frac{D_{c}-D}{D_{c}-1}\right)}^{w}$$

The parameters *D_c_* and “*w”* after from [16] were assumed to be equal to 1.2 and 6, respectively. The fracture process onset when *D* = 1, and when *D* = *D_c_*, complete split occurred. The shape of the dependence of β parameter on the damage indicator *D* described by (15) is linear for *w* = 1, but in general it is nonlinear.

## 4. Numerical Model

**Figure 6:**
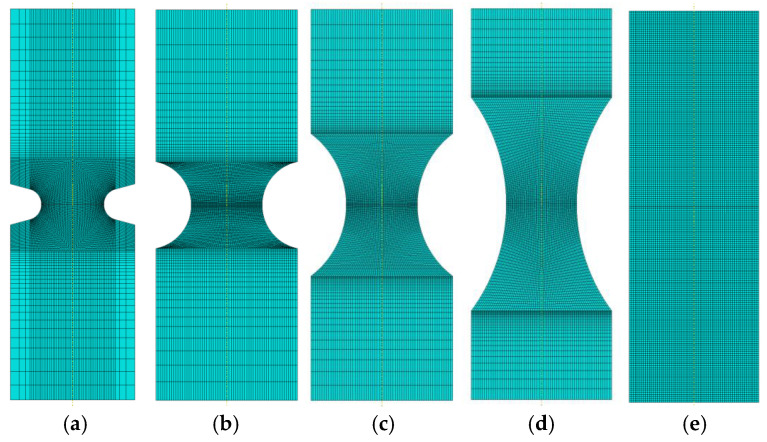
Geometry of the specimens tested in the program. R = 1.0 (**a**), R = 2.5 (**b**), R = 5.0 (**c**), R = 10.0 (**d**), smooth round (**e**).

The geometries shown in Figure 6 were adopted for the calculations.

The specimens were modeled in such a way that the minimum diameter (critical section) was always a circle with a diameter of 4 mm.

The calculations were performed in Abaqus/Explicit version 6.12-2, in the Linux environment. The VUMAT procedure was used for modeling the material, as it allows the user to program the effect of the stress triaxiality on the development of plasticity and failure of the element.

The geometry was modeled using CAX4R linear axisymmetric elements. The critical cross-section for each geometry was filled with elements with a size of 2 mm/32 = 0.0625 mm. The step-in time was determined automatically by the Abaqus routine (version 6.12-2), based on the size of the smallest element.

As the plasticity theory used in the paper is valid under several assumptions, that is, the homogeneity and material isotropy, and the material is taken to be elastic–plastic with isotropic hardening, we applied isotropic hardening.

Using the existing symmetries, a quarter of the geometry shown in Figure 6 was always modeled.

**Figure 7:**
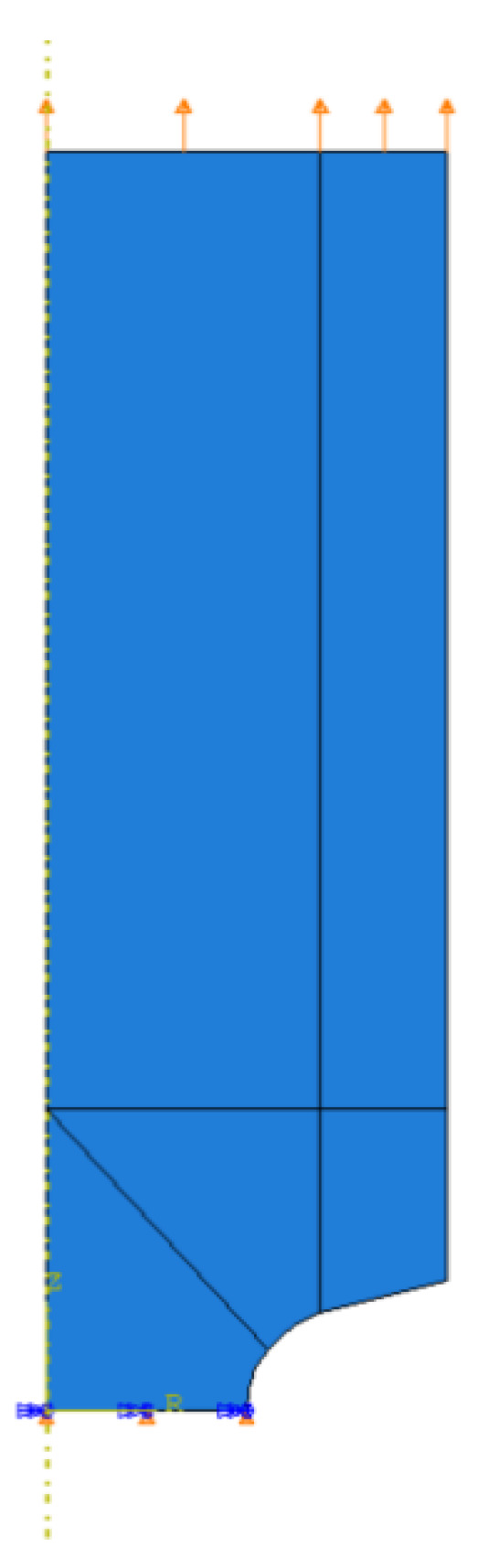
Boundary conditions.

The uniform displacement was applied to the upper edge of each model (Figure 7).

## 5. Tensile Tests

**Figure 8:**
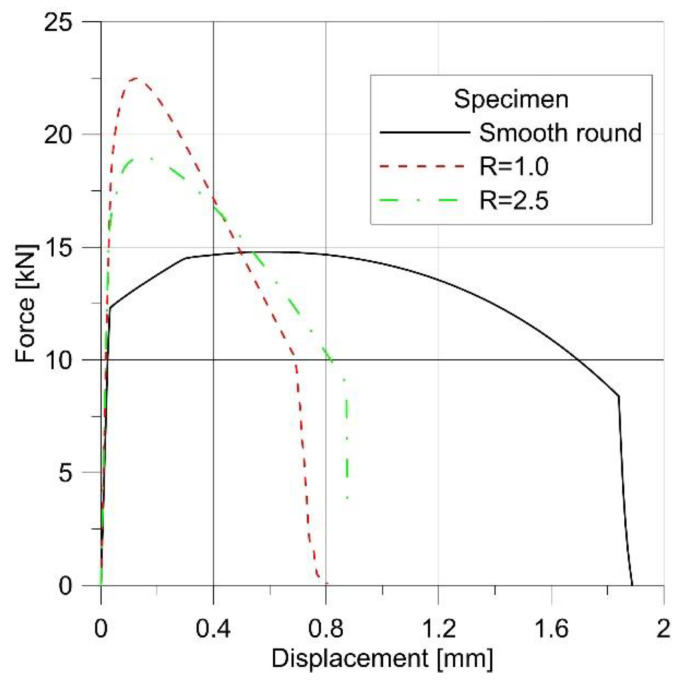
Force–displacement plots for different geometries.

The reference state for simulating the tensile test was a cylindrical specimen with a diameter of 4 mm (Figure 6e). The result of the simulation was the force-displacement plot (Figure 8), and the distribution of the effective stress and the parameter *D*.

**Figure 9:**
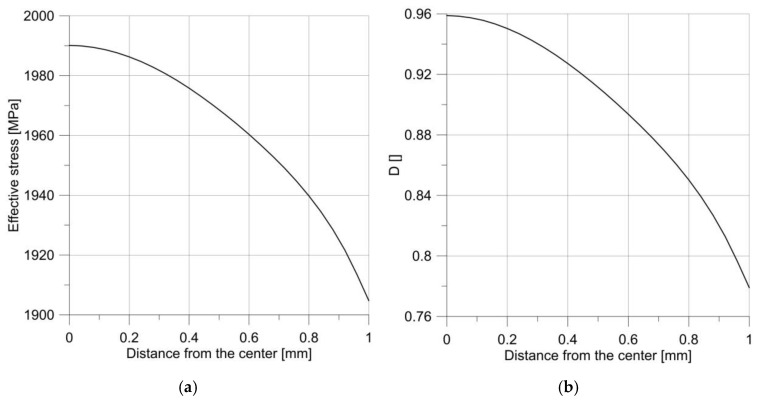
Distribution of effective stress (**a**) and parameter *D* (**b**) just before the fracture initiation of the smooth round specimen.

**Figure 10:**
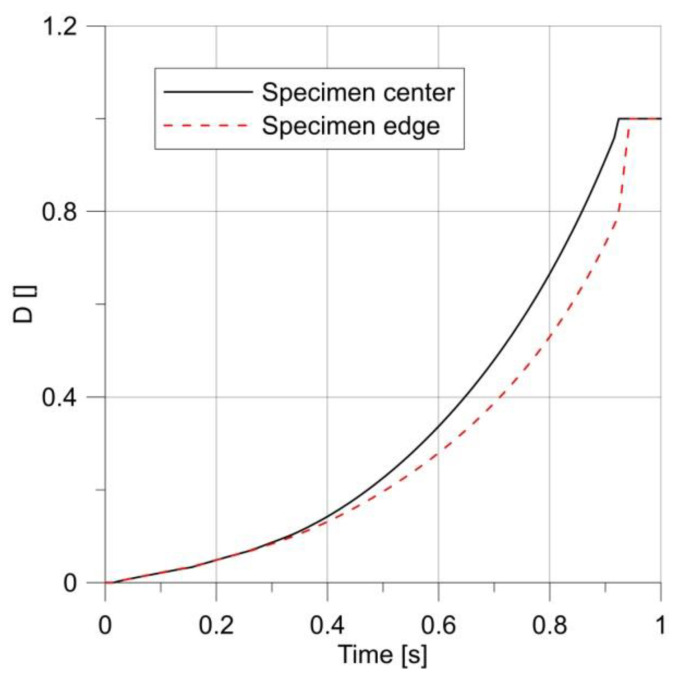
Changes in the *D* parameter over time for a smooth round specimen.

From the distribution of the effective stress and the *D* parameter, it can be seen (Figure 9) that in a smooth round bar the damage process began in the specimen axis. The result of this behavior on the specimen surface was the appearance of a cup-and-cone. Figure 10 shows how the parameter *D* changed at the edge and in the center of the specimen. It shows that at low loads the failure parameter had a constant value in the cross-section, whereas increasing the load caused a slightly faster increase in *D* in the specimen center.

Introducing a small-radius notch influenced the behavior of the sample during the test. Loading the specimen with a notch of the radius of 1 mm allowed us to obtain a much higher maximum force (Figure 8), and the specimen fractured with much less displacement (strain). This was associated with the change in the level of triaxiality in the specimen.

**Figure 11:**
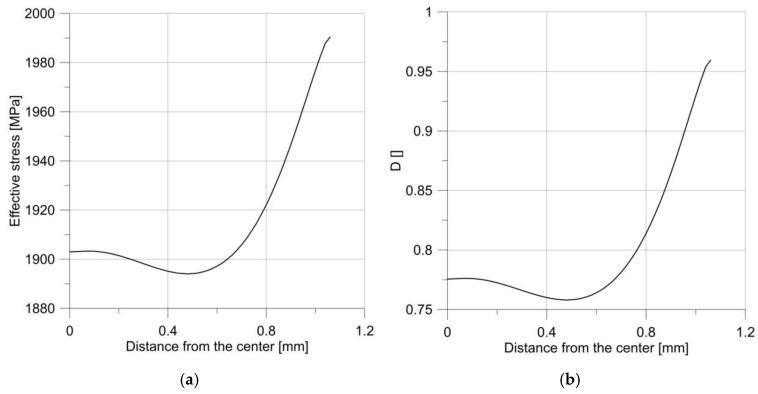
Distribution of effective stress (**a**) and parameter *D* (**b**) just before the moment of failure of the specimen with the notch R = 1 mm.

**Figure 12:**
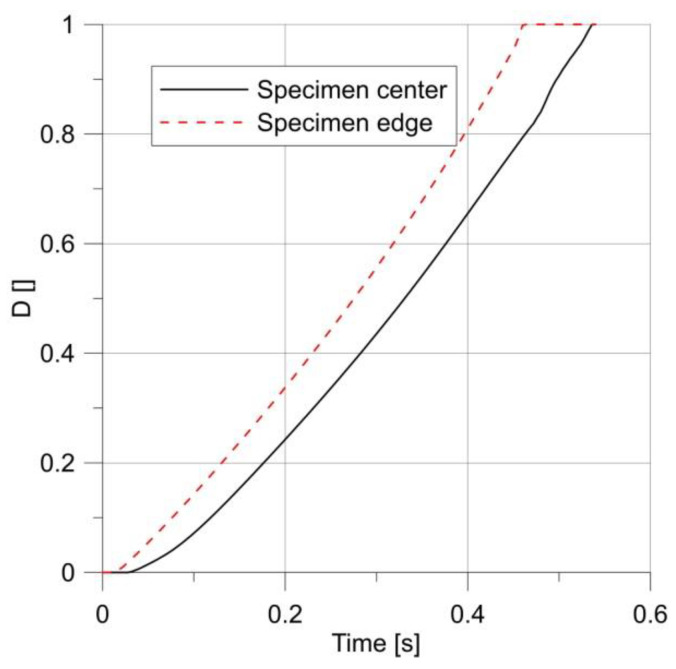
Changes in parameter *D* over time for a specimen with a notch R = 1.0 mm.

The changes in effective stress and the *D* parameter are shown in Figure 11. The presented results show that the failure process started from the notch root. The changes in the *D* parameter in the specimen center, and the notch root during loading, as shown in Figure 12, proved that the increase in the parameter describing the level of damage was much higher compared with the smooth round specimen. Moreover, from the beginning of loading, this process developed more intensively in the notch root.

**Figure 13:**
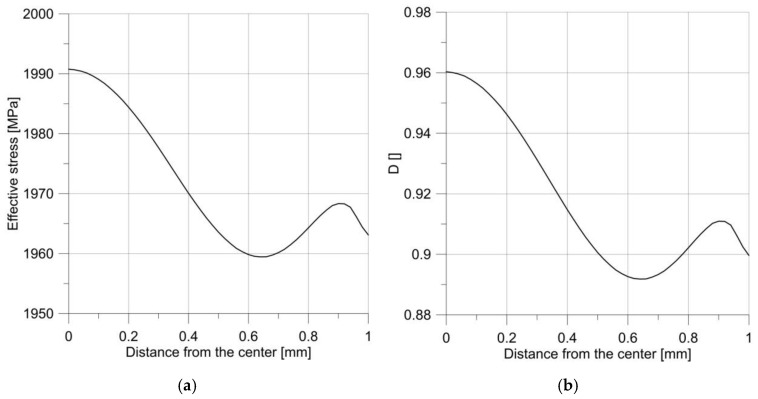
The distribution of effective stress (**a**) and the parameter *D* (**b**) just before the moment of failure of the sample, with the notch R = 2.5 mm.

It may seem that such a situation will be true for every notch; however, increasing the radius to only 2.5 mm resulted in a change in the behavior of the specimen. As it is clear to see in Figure 6, the maximum force obtained during the tensile test was much lower, but the critical displacement was much larger. The diagrams of effective stress and the *D* parameter were much more interesting (Figure 13).

It can be seen that the courses of the mentioned parameters were irregular, and the maximum was not at the notch root, but in the specimen center.

**Figure 14:**
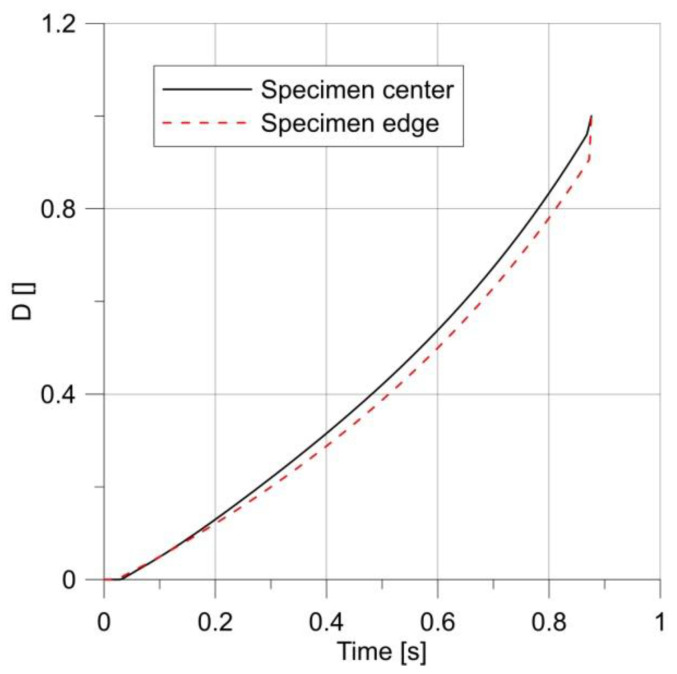
Changes in parameter *D* over time for a specimen with a notch R = 2.5 mm.

The plot comparing the *D* parameter changes in the center of the specimen and at the notch root (Figure 14) resembled that of a smooth sample (Figure 10).

It was interesting that these changes in the behavior of the specimens subjected to monotonic loading translated into the behavior of cyclic loading.

## 6. Fatigue Tests

**Figure 15:**
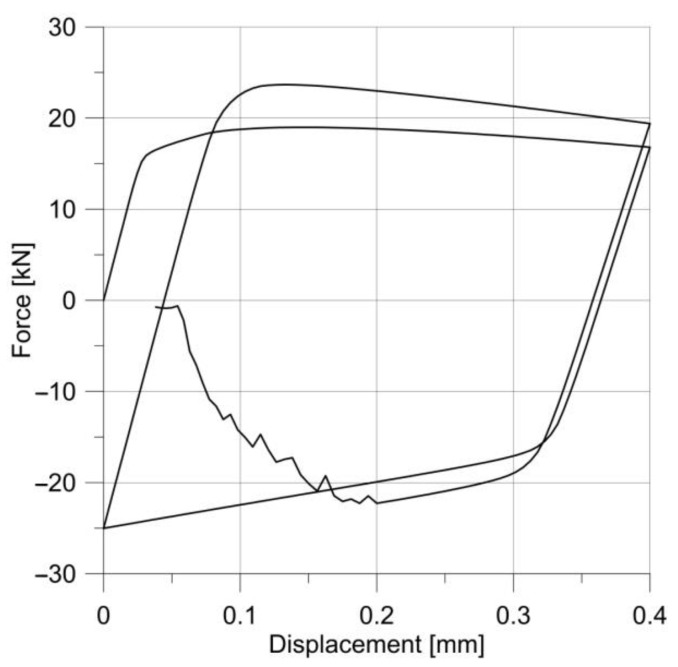
Changes in the stress–strain hysteresis loop for a specimen with a notch R = 2.5 mm.

All specimens were loaded with repeated stress cycles. The first sample for fatigue testing was a specimen with a notch radius of 2.5 mm, loaded with a sinusoidal variable load with an amplitude of 0.2 mm and a mean displacement in a cycle of 0.2 mm. The material response for cyclic loading is shown in Figure 15.

**Figure 16:**
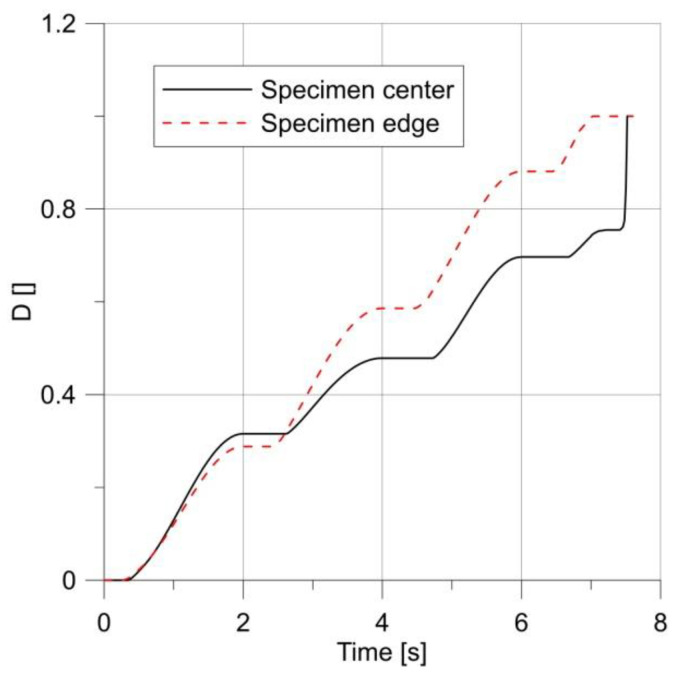
Changes in parameter *D* under load.

The changes in the *D* parameter during loading are shown in Figure 16. It can be seen that from the initial stage of loading until the first maximum, the specimen behaved in accordance with the results obtained for the monotonic loading, but the first unloading caused a change in the behavior of the specimen, and the maximum shifted towards the notch root.

**Figure 17:**
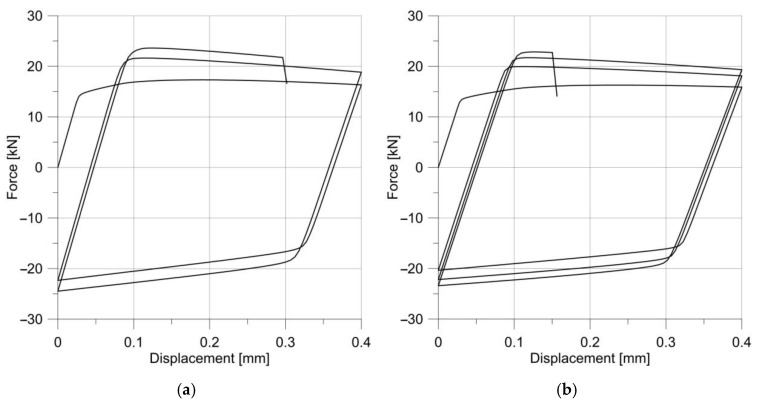
Changes in the stress–strain hysteresis loops for specimens with notches R = 5.0 mm (**a**) and R = 10 mm (**b**).

The tests were repeated for specimens with much larger notch radii, i.e., 5 and 10 mm; the load amplitude remained the same. The material response is shown in Figure 17.

**Figure 18:**
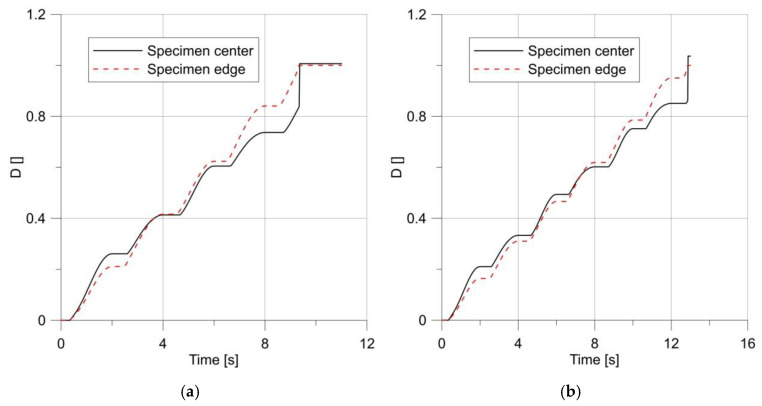
Changes in parameter *D* under a load, for specimens with notch radius R = 5.0 mm (**a**) and R = 10 mm (**b**).

For these specimens, the loading process ended with a fracture initiated in the notch root (Figure 18).

The presented results showed that increasing the radius of the notch root improved the fatigue strength, with a greater number of load cycles. During the fatigue test, at some point the damage of the material, initially developing faster in the specimen center, began to dominate at the notch root [27,28]. The change in the place of damage dominance depended on the notch root radius; the later it occurred, the greater the notch radius was. Unfortunately, the behavior of the specimen similar to this under a under a monotonic load was not obtained. In an attempt to obtain such behavior during the fatigue loading, tests were carried out on a smooth round specimen.

**Figure 19:**
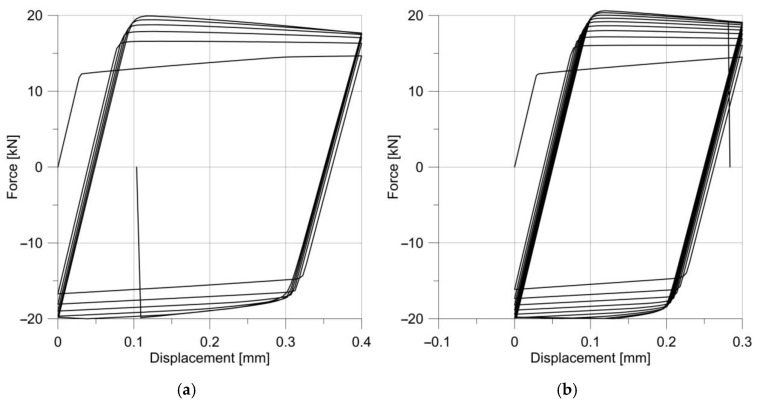
Change in the stress–strain hysteresis loop for smooth round specimens with a regular load (**a**) and a load decreased by 25% (**b**).

Two levels of loading were used: the standard load used for other specimens, and a load decreased by 25%. Reducing the load increased the number of cycles from 5 to 9 (Figure 19).

**Figure 20:**
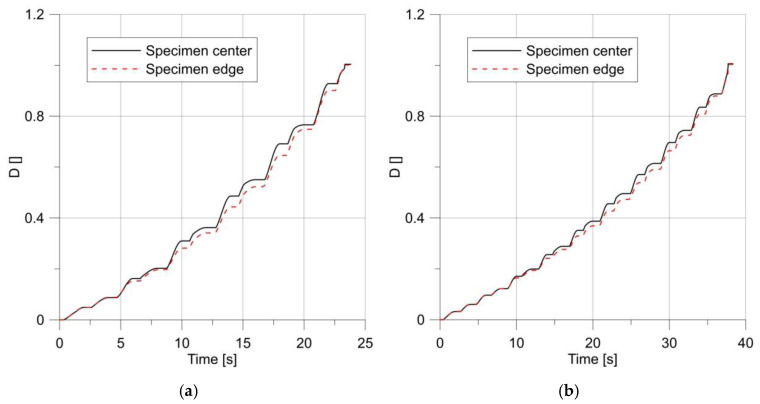
Changes in parameter *D* for a smooth round specimen with a regular load (**a**) and a reduced load (**b**).

Changes in the damage level at two characteristic points are shown in Figure 20.

**Figure 21:**
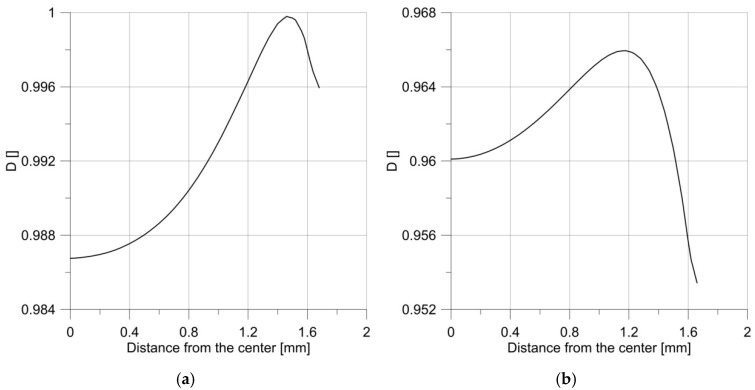
Distribution of the *D* parameter just before the crack initiation for the regular load (**a**) and the decreased load (**b**).

Parameter *D*, in both cases, had a higher value at the specimen center during almost the entire loading time, but in the final phase the maximum damage position changed, probably due to the influence of the neck curvature. Determining the location of the *D* maximum required us to trace the distribution of the *D* parameter at the last moment before the crack initiation (Figure 21). In the case of a smooth round specimen subjected to ultra-low-cycle fatigue, the crack initiation occurred near the outer surface, but not at the root of the notch produced by the neck. This was confirmed by numerous examples of fracture surfaces obtained during the research [29,30,31].

**Figure 22:**
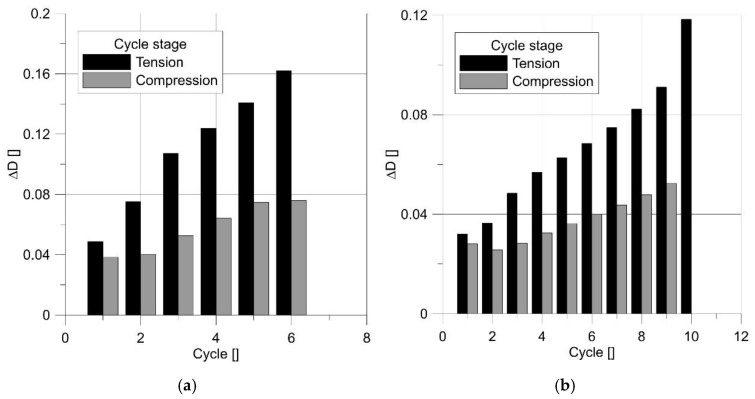
Increment of parameter *D* for subsequent cycles.

Figure 22 shows how the rate of material damage accelerated with the cycle. This diagram also reveals the asymmetry of the damage accumulation. Despite the assumption of the symmetry of behavior concerning tension and compression, a greater increase in damage was recorded in the case of tension.

Similar behavior was also observed with the notched specimens.

## 7. Discussion and Conclusions

This paper used the Wierzbicki–Bai model to analyze the damage process of elements subjected to monotonic and fatigue loads. Smooth round and notched specimens were analyzed. For the material of the specimens, Inconel 718 was selected with the assumption of isotropic hardening. As this assumption could be too rough, further investigations are necessary on the combined-material hardening rule. Nevertheless, the results showed that the Wierzbicki–Bai model captured the differences in the behavior of the specimens with different geometries, and the development of the damage in the analyzed elements. In a smooth round specimen subjected to tension, the greatest increase in damage occurred in the center of the sample, demonstrating that they are harder to fracture than notched specimens. The introduction of the small radius notch to the specimen geometry in the tensile specimens caused a crack initiation in the root of the notch; however, a sufficiently large increase in the notch radius caused the fracture process to initiate in the specimen center, as in the case of a smooth round specimen. As a result, the smooth specimens fractured more dynamically than the notched ones that we could observe during the simulation. The results proved that it was possible to locate the hotspot of the specimen: the place where a combination of high-effective stress, stress triaxiality, and the Lode parameter was favorable for the initiation of cracks. This information is of great importance for engineers, with regard to how they can change the geometry of machine members to improve their strength.

**Figure 23:**
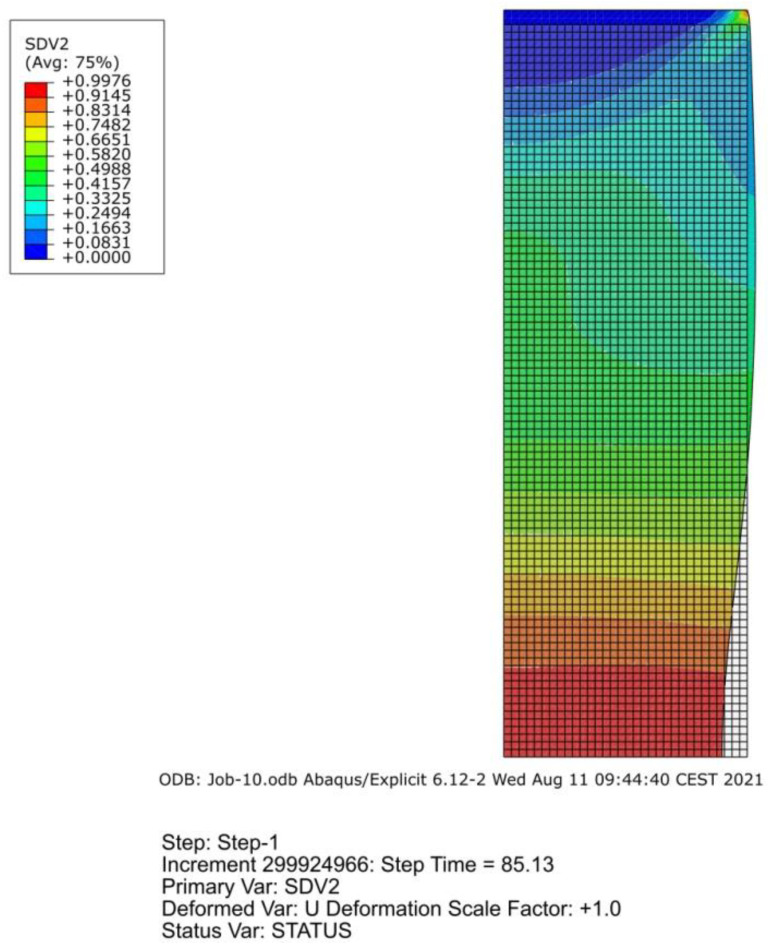
Smooth specimen shape at the final stage.

The situation was completely different for fatigue loads. In the case of the specimens with a notch, a fracture began at the root of the notch, regardless of the size of the radius. In the case of smooth round specimens, the crack initiation did not occur at the specimen center, as in the case of monotonic loading. The initiation site was close to the outer surface, that is, near the root of the forming neck. Such a state is confirmed by numerous experimental studies. The position of the crack initiation site in the smooth round specimen was to some extent affected by the load level; unfortunately, it was not possible to obtain a state in which the fatigue crack initiation would occur in the center of the specimen. The probable cause of this was the notch influence. Even in the smooth specimens at the final stage, one can observe a notch, as is presented in Figure 23.

The increase in the damage of the specimens subjected to fatigue load was much more intense during a tensile stage in the cycle, even though the symmetry of the yield locus was adopted, but only when the Lode parameter was considered. This proved that the main source of the phenomenon was a different level of stress triaxiality for tension and compression. This result shows that ultra-low-cycle fatigue can be utilized to calibrate the value of the coefficient of stress triaxiality dependency on plasticity *c_η_*. The most popular calibration method so far has been to compare the results of the tensile test and the upsetting test; however, the ultra-low-cycle fatigue test would be faster and less expensive, as it uses only a single specimen.

## Figures and Tables

**Table 1:** Coefficients of the fracture locus (source: own computations).

*N*=	−0.4773	0.319	−0.7304
	0.6683	−0.5705	1.5615
	−0.2423	0.514	−1.8897
	−0.0526	−0.3344	1.4992

## Data Availability

No new data were created or analyzed in this study. Data sharing is not applicable to this article.
